# Roles of neuroligins in central nervous system development: focus on glial neuroligins and neuron neuroligins

**DOI:** 10.1186/s12967-022-03625-y

**Published:** 2022-09-10

**Authors:** Xing Liu, Fuzhou Hua, Danying Yang, Yue Lin, Lieliang Zhang, Jun Ying, Hongguang Sheng, Xifeng Wang

**Affiliations:** 1 Department of Anesthesiology, The Second Affiliated Hospital of Nanchang University, Nanchang, 330006 Jiangxi China; 2 Key Laboratory of Anesthesiology of Jiangxi Province, 1# Minde Road, Nanchang, 330006 Jiangxi People’s Republic of China; 3 Department of Anesthesiology, The First Affiliated Hospital of Nanchang University, Nanchang, 330006 Jiangxi China

**Keywords:** Neuroligins, Neurexin, Neuroglia, Synaptic transmission, Synaptic plasticity, Neurodevelopmental disorders

## Abstract

Neuroligins are postsynaptic cell adhesion molecules that are relevant to many neurodevelopmental disorders. They are differentially enriched at the postsynapse and interact with their presynaptic ligands, neurexins, whose differential binding to neuroligins has been shown to regulate synaptogenesis, transmission, and other synaptic properties. The proper functioning of functional networks in the brain depends on the proper connection between neuronal synapses. Impaired synaptogenesis or synaptic transmission results in synaptic dysfunction, and these synaptic pathologies are the basis for many neurodevelopmental disorders. Deletions or mutations in the neuroligins genes have been found in patients with both autism and schizophrenia. It is because of the important role of neuroligins in synaptic connectivity and synaptic dysfunction that studies on neuroligins in the past have mainly focused on their expression in neurons. As studies on the expression of genes specific to various cells of the central nervous system deepened, neuroligins were found to be expressed in non-neuronal cells as well. In the central nervous system, glial cells are the most representative non-neuronal cells, which can also express neuroligins in large amounts, especially astrocytes and oligodendrocytes, and they are involved in the regulation of synaptic function, as are neuronal neuroligins. This review examines the mechanisms of neuron neuroligins and non-neuronal neuroligins in the central nervous system and also discusses the important role of neuroligins in the development of the central nervous system and neurodevelopmental disorders from the perspective of neuronal neuroligins and glial neuroligins.

## Introduction

Signals transmitted in synapses are the basis for information processing in the brain, and neurons form a network of intercommunication and communication through synapses. In this vast network of information, synapses play a role in transmitting, processing, and refining information. In recent years, a class of proteins containing cell adhesion molecules (CAMs) has been identified in synapses. Neuroligins (NLs) and neurexins (NRXs) are considered to be well-characterized cell adhesion molecules and, they are also molecules with specific synaptic functions [1, 2]. Specific synaptic structures are formed through the interactions between these CAMs. Specifically, to ensure proper synaptic function, specific axons need to be connected to specific dendrites [3, 4]. In this context, these CAMs can meet the need for synaptic communication by recruiting specific neurotransmitter receptors.

The current study shows that NLs are actively involved in regulating synaptogenesis and synaptic function. Their trans-synaptic interactions with presynaptic NRXs are the basis for their complex functions. The specific recognition and connection between axons and dendrites rely on the complex recognition code between NLs and NRXs [5, 6]. In addition to this, NLs have been widely reported to be associated with the regulation of *N*-methyl-d-aspartate receptor (NMDAR) and alpha-amino-3-hydroxy-5-methyl-4-isoxazole propionic acid receptor (AMPAR) functions [7, 8], and to be involved in the recruitment of synaptic proteins and the regulation of synaptic plasticity [9–11]. This proper synaptic contact and synaptic plasticity ensure that the brain functions properly. Since a range of neurodevelopmental disorders such as Autism Spectrum Disorder and Schizophrenia are closely related to synaptic dysfunction [12]. Consequently, NLs-related synaptic dysfunction is thought to underlie brain development and cognition. With the intensive research on the expression of various cell-specific genes in the central nervous system (CNS) in recent years, the function of glial NLs in the CNS has received more and more attention. In this review, we focused on the molecular mechanism of neuronal NL and non-neuronal NL in CNS from the aspects of subcellular localization, structural changes, and ligands of neuroligins, and construct a more complete conceptual framework for the role of neuroligins expressed in various types of glial cells. Meanwhile, we summarize the roles of neuronal NLs and glial NLs in synaptic development, synaptic transmission, and synaptic connections. We also focused our attention on the pathophysiological role of glial NLs and neuronal NLs in neurodevelopmental disorders.

## General characteristics of neuroligin

Years before neuroligins were discovered, neurexins, a presynaptic transmembrane receptor that interacts with them, were identified as a candidate molecule encoding inter-neuronal communication [13, 14], and it can induce the release of large amounts of neurotransmitters from nerve endings to regulate various complex functions of synapses (reviewed in [15]). The realization of complex synaptic functions is not only dependent on the presynaptic membrane, which releases neurotransmitters, and the postsynaptic membrane, which responds to neurotransmitters. The complex protein interactions that take place in the synaptic gap (the space between the presynaptic and postsynaptic membranes) are also critical to the realization of synaptic function. The binding of neuroligin and neurexin then plays a key role in the dynamic regulation of these complex sets of protein interactions. Specifically, neurexins of the presynaptic membrane extend their extracellular structural domains into the synaptic gap and bind to the postsynaptic membrane ligand neuroligin to form trans-synaptic bridges (other postsynaptic membrane ligands of neurexin are beyond the focus of this review) (reviewed in [16]). Activity-dependent shearing of neuroligin and its ligands, changes in structural conformation in allosteric regulation, or competition for overlapping binding sites between them will affect their binding [17–19], and it may be these changes that contribute to the function of neuroligin.

### Structure and subtypes

Neuroligin is a single-pass type-I transmembrane protein. Humans express five NLs genes, including four family members common to mammals (NL1, 2, 3 and NL4) and an additional homologue in the human Y chromosome [12, 20–22]. In general, they have structural similarities in amino acid sequences, but to a certain extent they have the specific conserved structural domains they have large extracellular acetylcholinesterase-like structural domains, and these exocytotic structural domains are localized to the postsynaptic membrane [23]. In addition, they have cytoplasmic tails that bind scaffold molecules (e.g., postsynaptic density protein 95, gephyrin, collybistin) [24, 25], and a specific transmembrane domain exists in each region to separate these extracellular acetylcholinesterase-like domains from the cytoplasmic tails [26, 27]. Each NL contains two different alternative splice sites and generates different extracellular structural domains by insertional alternative splicing [28]. These alternative splice sites can influence the specificity of trans-synaptic adhesion interactions [6, 28]. In particular, NL 1 contains a splice site at position A (SSA) that can carry a 20 or 40 a.a. insert, and an additional splice site at position B (SSB) to carry a 9 a.a. glycosylated splice inserts [16]. Short exons contained at these two sites give rise to four NL1 splicing variants (NL1(-), NL1A, NL1B, and NL1AB) [29]. Similarly, alternative splicing of NL2 at SSA can also generate two splicing variants (NL2(-) and NL2A). The different splice sites of NLs create a series of changes in the binding.

Affinity and synaptic induction activity of NRXs and NLs [6, 28], thereby affecting cellular functions including synaptic formation rate and synaptic plasticity [30, 31]. Therefore, splicing variants of NLs play a modulatory role in the NLs–NRXs complexes that regulate excitatory and inhibitory synapses. Notably, bridging of NL1 and NRX1α is thought to occur via Hevin secreted by astrocytes, which is critical for the formation and plasticity of thalamocortical connections in the developing visual cortex [37]. This also suggests that NLs are also closely associated with glial cells in the central nervous system.

### Subcellular distribution

Different isoforms of neuroligins have their unique subcellular localization. Initially, immunostaining and biochemical analysis showed that NL1 is localized at glutamatergic (excitatory) synapses; NL2 is located at GABAergic (inhibitor) synapses, and NL3 can be localized at both excitatory and inhibitory postsynapses [23, 32–34]. Compared to the above NLs family, little is known about NL4. Unlike NL1-3, NL4 is poorly preserved evolutionarily (58% is preserved from humans to mice). Human NL4 is located after glutamatergic synapses, whereas mouse NL4-like is located after glycinergic synapses [35, 36], which limits the conclusion that mouse models are used for human cells [22]. NL mRNAs display unique expression profiles in a region-, cell-type-specific manner [37]. By chromogenic and fluorescent in situ hybridization, Uchigashima et al. found that NL1 mRNA had the highest signal in the hippocampus compared to NL2 and NL3, while expression was weak in all other regions of the CNS. In contrast, NL2 mRNA is abundantly expressed in both the hippocampus and olfactory mitral cell layer and cerebellar Purkinje cell layer. As for NL3 mRNA, it is expressed in various parts of the CNS, but its peak level can be observed in the hippocampus [37]. Consistent with the mRNA expression pattern, NL3 is expressed throughout the brain, with higher levels in the hippocampus, neocortex, striatum, and brainstem and lower levels in the thalamus and cerebellum (reviewed in [38]). Because of the unique role of NL3 splicing variants in regulating synaptic function, subcellular localization of NL3 splice isoforms becomes important. Regrettably, to date, there seem to be no studies on the subcellular localization of endogenous NL3 splicing variants, except for one article in which NL3 regulates inhibitory synaptic transmission in a splice subtype-dependent manner and demonstrates that expression of NL3 splice variants is highly expressed in hippocampal CA1 pyramidal neurons [39].

## The mechanism of neuroligin

### Dimerization

Since the extracellular structural domain for NL exhibits great similarity to the structure of acetylcholinesterase protein [29], it has been inferred that NLs are as prone to dimer formation as cholinesterases. Indeed, intracellular neuroligin naturally exists in the form of dimers [40]. When mutations in the acetylcholinesterase-like extracellular structural domain of NLs produce neuroligins mutants, they reduce the synaptic activity caused by biochemical dimers of NLs, as well as their adhesion to NRXs [41]. After the existence of NLs dimers was established, a large number of studies began to focus on the role of dimerization of NLs in the overall synapse formation process. It has been shown that it is neuroligins dimers (and not neuroligins monomers) that are selectively transported to the cell surface, implying that dimerization of neuroligins is a necessary form for their involvement in transport [42]. Not only that, the aggregation of neurexin in the early steps of differentiation of axonal segments into presynaptic terminals is completely dependent on the presence of neuroligins in the dimerized state [41, 43]. These data suggest that the dimerization of neuroligin is an indispensable step in the coordinated assembly of synapses. In addition to the above-mentioned homodimerization, there is also heterodimerization between neuroligin. It was found by coimmunoprecipitation that NL3 can heterodimerize with NL1 and NL2 to form NL1–NL3 and NL2–NL3 complexes [32]. Whether homodimeric or heterodimeric, this structural variation in neuroligins is an effective mechanism for ensuring synaptic diversity.

### Binding partners

In addition to the changes of neuroligins themselves involved in the process of synapse formation, their binding to ligands also plays a central role in synapse development. Here we will compare the mechanism of NLs binding to ligands in neuronal NLs and non-neuronal cells separately and explore the similarities and differences between them.

#### Neuron neuroligins

Chronologically, postsynaptic density protein 95 (PSD-95) was the first partner found to bind to Neuroligin. As a member of the membrane-associate guanylate kinase (MAGUK) family, PSD-95 contains three PDZ structural domains, one SH3 structural domain, and one GK structural domain starting from the N-terminal end [44]. Where NMDAR and potassium channels interact with the first and second PDZ structural domains, respectively, to regulate the functional properties of membrane proteins [45, 46]. The third PDZ structural domain of PSD-95 can then bind to the cytoplasmic C-terminus of Neuroligin to participate in the formation of the neuroligin–neurexin complex [47, 48]. Notably, consistent with the endogenous distribution of NLs, NL2 was co-recruited with PSD-95 and gephyrin (inhibitory synaptic scaffolding protein), whereas NL1 recruited only PSD-95 [33]. Since PSD-95 is a major component of glutamatergic excitatory synapses, and NL1 is similarly localized to excitatory synapses, many of the next studies focused on the physiological roles of NL-1 and PSD-95 in the glutamatergic signaling pathway. It is precisely because the PDZ domain of PSD-95 can bind to glutamate receptors and K^+^ channels, that there may be the aggregation of corresponding neurotransmitter receptors and ion channels at the intercellular junction site of neuroligin-neurexin, to play the role of NRX–NL axis in the regulation of synaptic activity. Studies have shown that PSD-95 can recruit NL1 to excitatory synapses and transfer NL2 from inhibitory synapses to excitatory synapses to change the ratio of excitatory and inhibitory synapses [33, 49]. In addition, Dean et al. found that recruitment of neurexin mutants lacking cytoplasmic tails containing PDZ domains could not lead to effective presynaptic differentiation [41]. All these indicate that presynaptic or postsynaptic differentiation is largely dependent on the interaction of scaffolding proteins with NLs or NRXs.

In contrast, for inhibitory synapses such as glycinergic and GABAergic synapses, Gephyrin becomes a major component of their postsynapses and plays an important role in the postsynaptic localization of these two types [50]. At GABAergic synapses, NL2 is co-localized post-synaptically with Gephyrin. The binding domain of NL2 with gephyrin is conservative in all neuroligin subtypes, which means that NL1, NL2, and NL3 can bind well with gephyrin [25]. This indicates that the specific role of NL2 in inhibitory synapses does not depend solely on the binding with gephyrin. Poulopoulos et al. also showed that NL2 has a specific binding partner different from NL1 or NL3: collybistin. NL2 can interact with collybistin to induce the targeted delivery of gephyrin-collybistin to the plasma membrane [25]. This certainly provides evidence for a specific role of NL2 at inhibitory synapses. Synaptic scaffolding molecule (S-SCAM), on the other hand, exists with a structural domain similar to PSD-95 that interacts with NMDAR subunits and neuroligin [51]. S-SCAM can bind to neuroligin through two different structural domains, PDZ- and WW-structural domains [52]. Unlike PSD-95, S-SCAM is present in both excitatory and inhibitory synapses. Moreover, the PDZ-binding domain and WW-binding domain are conserved in all neuroligin subtypes, so it is easy to see that S-SCAM can bind to and function in all neuroligin subtypes.

In comparison, NL3, the only NL subtype localized in excitatory and inhibitory synapses [23, 32–34], has long been thought to exist as only one presynaptic binding partner, NRX, whereas a recent study identified presynaptically-expressed type IIA receptor protein tyrosine phosphatase delta (PTPδ) as a selective ligand for NL3 [53]. As presynaptic hub proteins, it is similar to NRX in generating different subtypes by selective shearing of microexons [54]. Yoshida et al. showed that PTPδ, which lacks mini-exon B, can interact with NL3 in trans-synapse. Further structural analysis also revealed that PTPδ and NRX1β may compete for binding to NL3; interestingly, this competitive binding is only present in NL3 and not in the remaining NL subtypes, and the R451C mutation in Nlgn3 can block this noncanonical NL3-PTPδ signaling [53]. This new finding brings a new pathway of synaptic tissue interaction and also provides a new way of studying the role of NL3 in the regulation of synaptic function in non-neuronal cells, i.e., what role the interaction between NL3 and NRX and PTPδ, which are different signaling pathways, plays in the regulation of synaptogenesis or synaptic function in non-neuronal cells.

#### Non-neuronal neuroligin

Like neuronal NL, non-neuronal NL also relies on the NRX–NL axis to play a significant function in the central nervous system. However, unlike neuronal NL, PSD-95, an important binding partner of NL at the postsynapse, does not seem to be located in non-neuronal cells [44]. Therefore, signaling between non-neuronal NLs and NRXs is necessarily different from the neuronal NRX–NL axis. In the CNS, non-neuronal glial cells account for approximately half of the total cell population and play a large role in the formation of CNS systems and functions [55]. Thus, in the following, we focus on the perspective of glial cells.

Indeed, Scheiffele et al. found through in vitro studies that when NL1 is expressed exogenously in HEK293 cells, non-neuronal HEK293 cells can form heterologous connections with their co-cultured neurons [56]. Not only that, but non-neuronal HEK293 cells can also trigger the formation of presynaptic specialization at heterologous junctions [56]. Similarly, Chubykin et al. found that the artificial synapse induced by NL1 could even show normal synaptic morphology, including presynaptic active regions, docked vesicles, and even usually postsynaptic density [57]. Of course, such exogenous expression of NL on synapses has all been shown to act by relying on its presynaptic ligand NRXs [33, 58].

In addition to the exogenous expression of NL mentioned above, the effect of endogenous expression of NL should not be neglected as well. With the improvement of cell purification techniques, techniques such as single-cell analysis of brain cells [59, 60], ACS sorting or cell-specific ribosome purification [61, 62], or acutely purified human astrocytes [63] have been used to delve into the cell type-specific transcriptome in CNS. Analysis of these data reveals that NL is widely present in astrocytes, schwann cells, and oligodendrocytes in the retina and spinal cord [64, 65], and not only that, the expression levels of NLs1–3 in astrocytes and oligodendrocytes may even be higher than in neurons [63]. This has forced us to focus on the role of glial cell NL in the development of the whole CNS. Existing studies mainly focus on the regulation of synapse or glial cell structure and function by the combination of glial NL and presynaptic NRX. This part will be described in detail below.

The above NL binding to presynaptic NRX plays a role in synaptic regulation through the NRX–NL axis. However, when studies are not limited to the NRX–NL axis, we can identify other possible mechanisms of NL in the signaling between glial cells and synapses. Recent studies have shown that the extracellular structural domain of NL plays an important role in postsynaptic interactions. For example, Budreck et al. found that NL1-specific recruitment of endogenous NMDAR was not dependent on PSD-95 because deletion of the C-terminal structural domain of NL1 did not prevent the formation of the NL1–NMDAR complex [66]. NL1 can determine the synaptic abundance of NMDAR through the interaction of its specific extracellular structural domain with the GluN1 subunit of NMDAR [66]. NL1 KO mice can exhibit lower NMDA current [67]. Moreover, researchers found that in transfected neurons, postsynaptic overexpression of NL1 can significantly increase synaptic density, but its binding with NRX is not necessary for this ability to increase synaptic density [68]. These studies have proved that there are some mechanisms by which NL1 directly binds to glutamate receptors to affect synaptic strength. This conflicts with this classical model that NL requires binding to synaptic receptors via scaffolding molecules such as PSD-95 and Gephyrin. In inhibitory synapses, NL2 binding to gephyrin and collybistin controls inhibitory synaptic maturation by regulating GABA receptor aggregation on neurons [25]. In contrast, recent studies have shown that the MAM structural domain-containing GPI anchor protein MDGA binds specifically to NL2 through its Ig structural domain and, in doing so, disrupts NL2–NRX interactions and negatively regulates inhibitory synapses [69]. Although the above NL1 and NL2 extracellular binding sites have not been identified, these data provide some potential evidence that NL expressed in glial cells lacking scaffold molecules such as PSD-95 and gephyrin can communicate with neurons.

## Neuroligin in glia

As more and more proteins that interact with NL are discovered, the understanding of NL signaling between synapses or between cells becomes more sophisticated. After summarizing the mechanisms of neuronal NL and non-neuronal NL in intercellular communication, we turned our attention to the role played by cell-specific NL in the CNS.

Since a certain review of glial neuroligins has been conducted recently [70], here we will summarize the results of these new studies. In this section, we focus on constructing a more complete conceptual framework for the role of neuroligins expressed in various types of glial cells.

### Disruption of glial NL damages the structure and function of glial cells and synapses

In the central nervous system, excitatory synapses mostly form at the junction between presynaptic axon terminals and postsynaptic dendritic spines [71], although, some inhibitory synapses may also be present [72]. Because the shape and volume in each part of the spine are closely related to synaptic signals, the number, density, and distribution of dendritic spines are crucial for synaptic signaling. For example, the volume of the spine head is positively correlated with currents such as the number of PSDs and AMPAR [73]. And the morphology of dendritic spines is maintained and sculpted by F-actin [74]. Not only that, but presynaptic actin can also form a reticulum around synaptic vesicles that can act as a scaffold for synaptic vesicle modulators [75] and organize postsynaptic neurotransmitter receptors (NMDAR, AMPAR) post-synaptically [76]. By examining the role of the cytoplasmic C-terminal structural domain (CTD) of NL1 in spine and synapse regulation, Liu et al. found that although NL1 is unnecessary for initial synapse formation, the CTD of NL1 can be activated through interaction with the spine-associated Rap GTPase-activating protein and subsequent activation of the LIM-domain activation of this signaling pathway leads to spine/synapse growth, and synaptic strength increases in the hippocampus [77]. NLs can also recruit wave regulatory complex (WRC) proteins to the cell membrane on the cell membrane to stimulate f-actin assembly and consequently affect synaptic transmission and growth [78, 79]. Studies of glial cells in recent years have enriched the role of glial cells in the CNS, instead of considering them only as support cells. They can extend branches and make dynamic contacts with neurons and synapses during CNS development [80], and the formation of these branches is also dependent on the f-actin cytoskeleton. This means that F-actin is relevant to both glial cells and synaptic signaling. When the structure of these branches is affected, both glial cells and neurons will be affected in a way that cannot be ignored [81].

In Drosophila, gliotactin plays an important role in the formation of the glia sheath and the establishment of the blood–nerve barrier. Because removal of mutants of gliotactin leads to disruption of glial wrapping, resulting in disruption and paralysis of the blood–nerve barrier [82]. In addition, Gilbert et al. found that NL 3 is a retinal homologue of gliotactin, and many glial cells (Schwann cells, retinal astrocytes, and spinal cord astrocytes) can express NL3 during the development of the peripheral nervous system (PNS) and CNS [20]. In the future, it will be very meaningful to understand the loss of NL expressed by different glial cells leading to CNS and the disruption of the function of each glial cell itself.

### Glia neuroligin in myelination

Myelin is the glial plasma membrane arranged in multiple layers and concentrically wrapped around axons, which can accelerate signal transmission and reduce the energy consumption of neurons [83]. In the PNS, NL1 is a component involved in myelin formation expressed by Schwann cells to promote the formation and maturation of nerve myelin [84]. Also, since NL1 expression on Schwann cells is increased during sensory neuron depolarization and signaling of Schwann cell-associated axons, NL1 can be used as a suitable marker for sensory Schwann cells [85]. In addition, previous studies on gliotactin, a homolog of NL3, demonstrated that gliotactin is required for myelin development in the Drosophila PNS [20]. Myelination in the PNS is relatively simple, with individual Schwann cells myelinating only a single axonal segment, and only large-diameter axons can be myelinated by Schwann cells [86]. In contrast, the pattern of myelination in the CNS is much more complex. Because myelination in the CNS follows a strict temporal sequence, axons in different regions are myelinated only at the correct time [87], a process that is heavily dependent on the differentiation of oligodendrocyte precursor cells (OPCs) to oligodendrocytes [88]. In the central nervous system, oligodendrocytes can express NL3, which was confirmed in the BarrasmRNA database [62]. Proctor et al. demonstratedthe existence of parallel axon-glial signals between neuronal axons neurexin and microglia dendrites NL3 by an in vitro rat section culture model [62]. Because in this model, cultured oligodendrocytes can come into contact with non-neuronal cells that exogenously express NLs conjugates NRX1α or NRX1β, and that oligodendrocyte differentiation is stalled at the immature stage when oligodendrocytes are cultured with NL1-ECD, a more competitive NLs conjugate than endogenous NLs. With this comes a decrease in the percentage of myelinated axons [62]. Limited by the fact that the above experiments were conducted in vitro, it is not easy to ensure that NRX–NL signaling is not affected, and in this model, neurotransmitter release may be compromised. Both oligodendrocyte differentiation and myelin formation are influenced by neurotransmitters, as glutamate released from axons can promote oligodendrocyte differentiation as well as myelin formation [89]. Considering the functional redundancy of NL, a more precise understanding of the role played by NL3 in myelin formation may require more accurate ultrastructural analysis to detect it, such as electron microscopic analysis.

### Neuroligins in astrocytes

Astrocytes are likewise highly complex glial cells in the central nervous system. They act as active participants in synapse development in the CNS, and they play a unique role in synapse formation, elimination, and synaptic plasticity [90]. Synapses are special structures between neurons, which are the basis of inter-neuron communication and the key to the proper connection of neuronal networks in the brain.

**Fig. 1:**
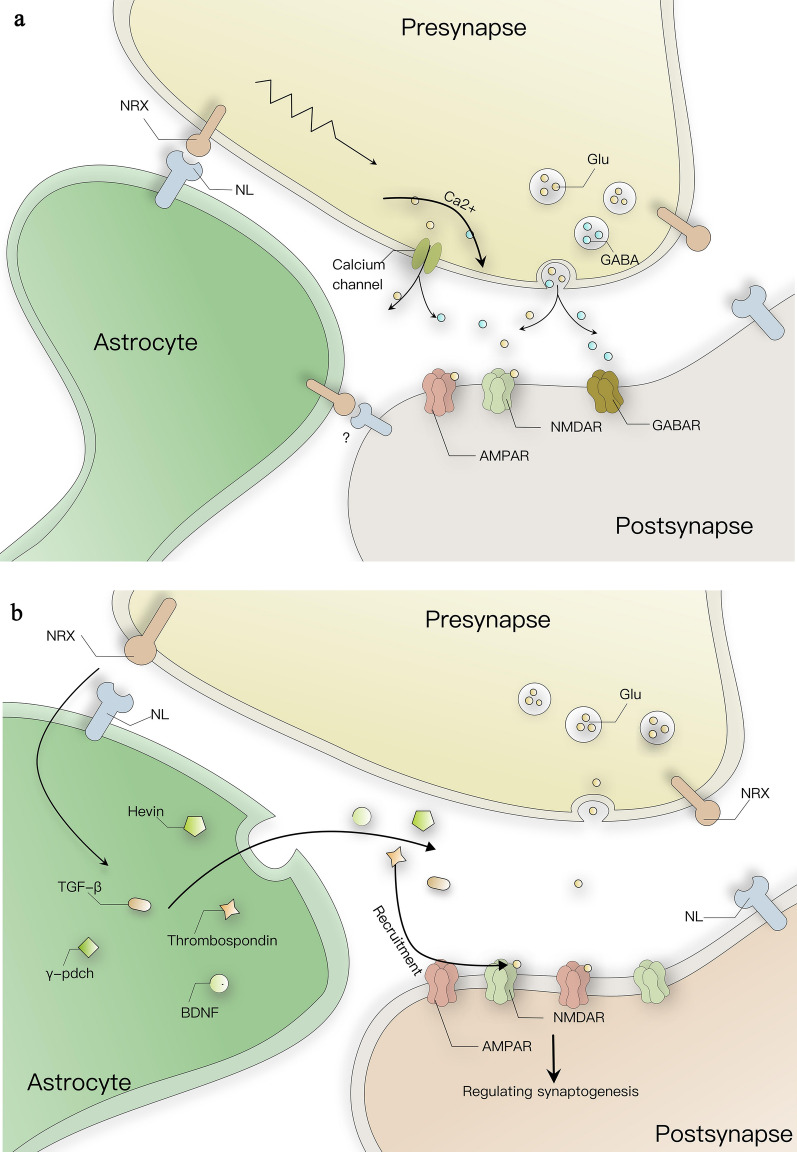
Neuroligins in astrocytes. **a** Schematic representation of neuroligin–neurexin intercontact. Astrocyte NLs can interact with presynaptic NRX. Their bidirectional transduction signals mediate the recruitment of calcium channels in the synaptic active zone. Neurotransmitters released by calcium channels influence synaptogenesis as well as function by binding to receptors on the synaptic surface. In addition to calcium channel recruitment, glutamate receptors such as NMDAR, AMPAR, and presynaptic vesicles are recruited to the synaptic active zone to mediate synaptogenesis and influence synaptic function. **b** Intercontact between astrocytes NL and presynaptic NRX may affect the expression of synaptogenic factors such as thrombin-reactive protein and SPARCL1/Hevin secreted by astrocytes. Neuroligin–neurexin intercontact resulting in elevated expression of synaptogenic factors can cause post-synaptic recruitment of AMPAR and NMDAR and affect synaptic generation. At the same time, these synaptogenic factors can also be targeted for release into the inter-synapse and participate in the regulation of synaptic function

According to the morphology and location, astrocytes can be divided into the protoplasmic astrocytes of the grey matter and the fibrous astrocytes of the white matter [91], where highly differentiated protoplasmic astrocytes can infiltrate into the surrounding nerve fibers. They finely control the process of synapse formation by secreting factors such as thrombospondin [92], Hevin [93], γ-protocadherins [94], TGF-β [95], brain-derived neurotrophic factor [96], etc., which induce glutamatergic synapse formation, increase the recruitment of postsynaptic AMPA receptors and NMDA receptors, and wrap neurons [97] (Fig. 1). And the fibrous astrocytes of the white matter are associated with myelinated axonal tracts. Therefore, it is clear that astrocytes play an important role in brain development and synaptic transmission. These roles depend in large part on the complex morphology and structure of astrocytes. The study of various synaptic modification molecules secreted by astrocytes and their compatibility with specific interaction partners provides directions for a better understanding of astroglial-neuronal signaling pathways and synaptic diversity in the CNS. NL expressed on astrocytes not only develops its own complex morphology and functional maturation through the role of neuron-expressed NRX, but also plays an essential role in synaptic development [104]. Communication between different cell types and neurons can be bridged by NRX–NL and play different roles. The understanding of these mechanisms highlights the importance of understanding the molecular regulation of interactions between various synaptic modification molecules, including NRX–NL.

In their study of the mechanisms underlying the establishment of the complex morphology of astrocytes, Stogsdill et al. found that the establishment of the complex morphology of astrocytes is closely related to NL [98]. Using this system, they demonstrated that in vitro, the morphogenesis triggered by astrocyte-neuron contact is inextricably linked to the roles of NL1-3 and neuronal NRX secreted by astrocytes. It is certainly a surprising result that, NL2 expressed by astrocytes controls the formation or maintenance of excitatory synapses within specific astrocytic regions in a cellular non-autonomous manner [98]. Whereas previous studies have shown that NL2 expressed by neurons are localized at inhibitory synapses [34] and is responsible for the regulation of inhibitory synapse formation and function [25, 99] (This section will be described in detail later).

The selective knockdown of astrocyte NL2 and whole-cell membrane clamp recordings of miniature excitatory and miniature inhibitory postsynaptic currents in cortical neurons revealed that the deletion of astrocyte NL2 significantly decreased the frequency and amplitude of miniature excitatory postsynaptic currents, along with an increase in the frequency of miniature inhibitory postsynaptic currents. This increase in the frequency of miniature inhibitory postsynaptic currents may be caused by an increase in the number of inhibitory synapses [98].

The connection between astrocytes and NL is much more than that. During synapse formation, Hevin secreted by astrocytes can induce synapse formation by bridging NRX–NL, and its antagonist SPARC can block the synaptogenic effect of Hevin. Previous studies have shown that the otherwise non-compatible presynaptic neurexin-1alpha NRX1α and postsynaptic NL1β in the developing mouse visual cortex can be connected via Hevin and thereby regulate the formation and refinement of glutamatergic synapses in the thalamo cortex [100]. Recent studies have also revealed the mechanisms by which Hevin, SPARC, and MDGAs (a negative regulator of synaptic development) interact with the extracellular matrix during synapse and neuronal circuit formation [101]. Fan et al. showed that the ratio of Hevin to SPARC and their competition for the binding sites on selective NL and NRX may promote or inhibit synapses, while the competitive binding of MDGA and Hevin to NL may determine the balance between excitatory and inhibitory synapses in CNS. Moreover, the combination of Hevin and collagen may connect NRX and NL bridges to the extracellular matrix to stabilize synapses [101]. αNRX1 and αNRX2 are also expressed in astrocytes [37], but how astrocyte-expressed NRX binds to postsynaptic NL is not yet known.

In conclusion, astrocyte-expressed NLs have a different mechanism of action in synaptogenesis than neuron-expressed NLs. On the one hand, bidirectional signaling of astrocyte NL and neuronal NRX adhesion may directly regulate synaptogenesis and function (Fig. 1a), while on the other hand astrocytes may regulate synaptogenesis by altering the expression of synaptogenic factors such as thrombospondin, SPARCL1/Hevin, or by targeted release to synapses. At the same time, bridging of NRX–NL to the extracellular matrix may stabilize synapses in a way that limits the diffusion of synaptic neurotransmitters (Fig. 1b).

## NLs in synapses

In recent years, there has been a growing interest in the NLs family in synaptic transmission and synaptic plasticity, as the integrity of synaptic transmission and the regulation of synaptic plasticity, are critical for the brain to process information. Simply stated, synaptic plasticity is when synaptic inputs change their strength due to prior activation. Long-term potentiation (LTP) and its counterpart long-term depression (LTD), as manifestations of synaptic plasticity, constitute the basic properties of most excitatory synapses in the CNS and are the basis for learning and memory [102]. In addition to learning and memory, long-term synaptic plasticity also plays an important role in areas including pain, addiction, neurodegenerative diseases, etc. In general, high-frequency afferent activity leads to an increase in NMDA receptor-mediated calcium inward flow, which increases intracellular calcium concentration and thus induces LTP [103]. NMDAR-dependent LTD is induced by weak activation of NMDARs (for example, due to modest membrane depolarization or low stimulation frequencies) and is thought to result from a smaller rise in postsynaptic Ca2+ than is required for LTP [102]. In addition to LTP and LTD, NL can also regulate presynaptic short-term plasticity (STP). STP can be divided into three categories: depression, facilitation, and augmentation/posttetanic potentiation (PTP). Normally, these three forms of plasticity can coexist within the same synapse [104]. The interactions between these forms of plasticity are then reflected by net synaptic strength [105]. It is well known that STP is closely related to calcium signaling, vesicle pools, postsynaptic transmitters, etc. [104, 106]. In synapses, NL can also affect these factors by binding to PSD-95, NRX, etc. Futai et al. measured the paired-pulse ratio (PPR) of the AMPAR-EPSC (which can be viewed as a form of presynaptic STP, usually inversely proportional to the presynaptic release probability) [106]. The PPR of PSD-95-transfected cells was significantly lower than that of untransfected neurons, suggesting that overexpression of PSD-95 increased the presynaptic release probability. It also confirmed the retrograde regulation of presynaptic glutamate release probability by PSD-95. Other than that, the sensitivity of presynaptic release to extracellular Ca2+ was also increased by postsynaptic overexpression of PSD-95 [107]. In other words, endogenous PSD-95 and NLG-mediated trans-synaptic signaling cascades are necessary for the retrograde modulation of the vesicle pool.

STP is also closely associated with each characteristic neuronal oscillatory activity in the CNS. It has long been known that different subtypes of specific interneurons in the brain can shape different rhythmic activities in the cortex, such that β oscillations are closely associated with stable activities such as preparation, while γ oscillations are more associated with dynamic activities such as motor seizures [108]. Abnormalities in specific rhythms in certain brain regions are commonly associated with human neurological or psychiatric disorders such as Parkinson’s disease [109] and schizophrenia [110]. In the cellular network model of Feng et al., model AMPA and GABA synapses exhibit short-term presynaptic plasticity between fast spiking inhibitory (FSI) and pyramidal cells (PN) [111]. Meanwhile, driving the network with short bursts of afferent activity drove relatively stronger γ, and driving with long duration bursts reduced γ power, but enhanced β. And when the STP mechanism is removed from the model, the above phenomenon disappears, which proves that STP plays a key role in the collaboration between β oscillations and γ oscillations [111]. Thus, the modulatory effects of NL on STP in the CNS may shape different oscillations in cortical circuits and in this way participate in the regulation of each behavioral state [112].

### NLs regulate excitatory synaptic transmission and synaptic plasticity

**Fig. 2:**
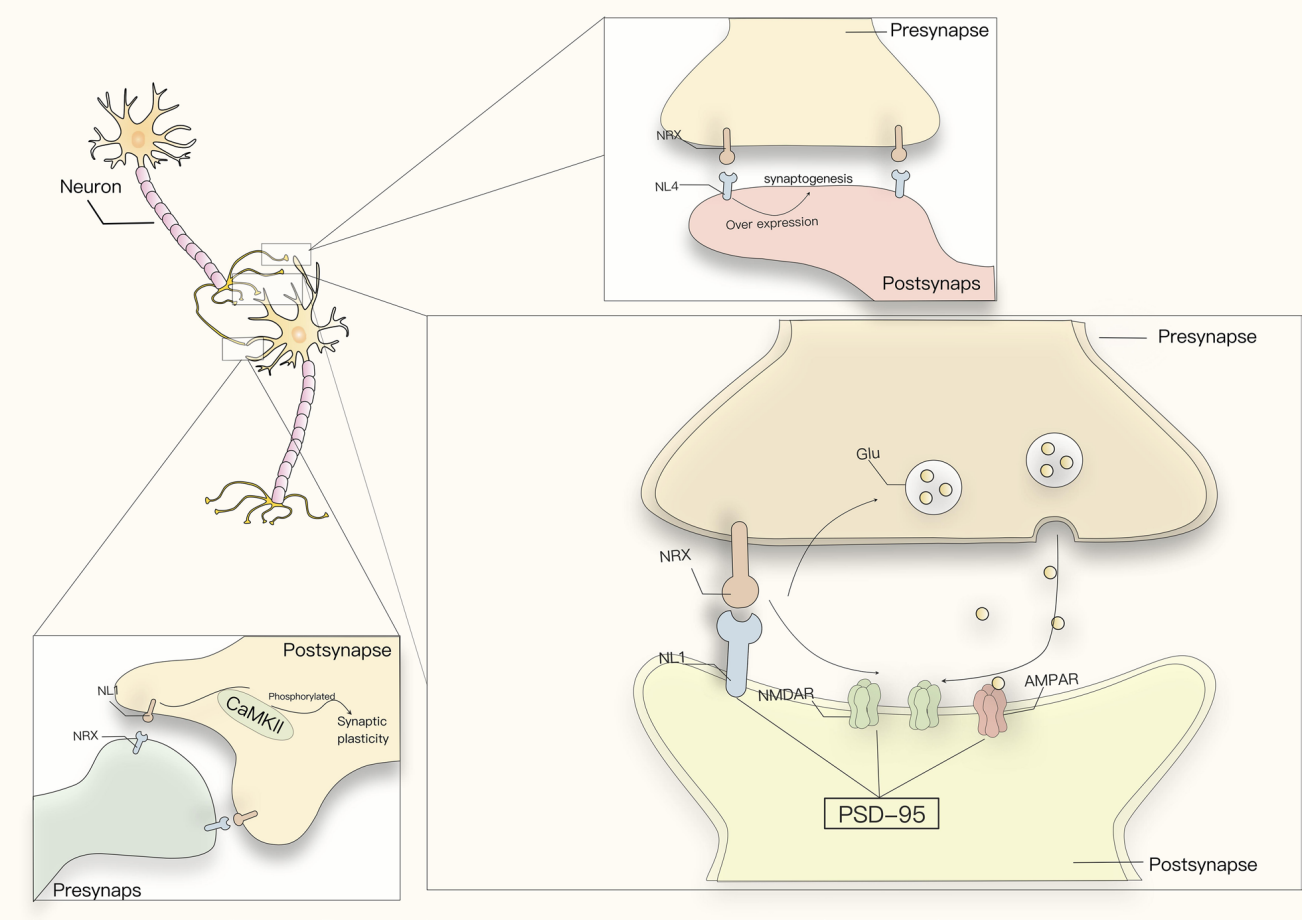
NLs in excitatory synapse. Neuroligin–neurexin interactions can affect the synaptic abundance of NMDAR, which in turn affects NMDAR-mediated synaptic transmission. They also affect the targeted release of glutamate, which is closely related to the efficiency of glutamate use. Postsynaptic NL1 can be phosphorylated by CaMKII and thus regulate synaptic plasticity

As described above, typically, NL1 is localized behind excitatory synapses to perform its function. Excitatory synapses are dominated by glutamatergic synapses. Both in studies of different regions of the brain and slices of neuronal cultures from different organisms, it is possible to conclude that there is a selective role for NL1 in glutamatergic synapses [8]. AMPAR mediate most fast excitatory synaptic transmission in the mammalian brain by interacting with the PDZ structural domain of PSD-95. Kalina et al. found a strong spatial correlation between the AMPAR nanodomain and the postsynaptic adhesion protein NL1, which was disrupted by the expression of truncated forms of NL1. To maintain the efficiency of high-speed synaptic responses, glutamate needs to be precisely released by the synapse in front of the AMPAR nanodomain, and synapses can optimize the use of glutamate through NL1-based trans-synaptic adhesion by controlling the alignment between the presynaptic release site and the AMPAR nanodomain with surprisingly high sensitivity [113] (Fig. 2). At the same time, knockdown or knockout of NL1 would result in a decrease in NMDAR-mediated postsynaptic currents, NL1 controls the synaptic abundance of NMDAR through the NL1-specific extracellular determinant cluster. Once this interaction is lost, NMDAR-mediated synaptic transmission will be impaired [66]. In contrast, NMDAR-mediated postsynaptic currents can be increased by overexpressing NL1 levels [114]. Neuroligins localized at excitatory synapses include NL4 in addition to NL1, Samuele et al. found that, unlike mouse Neuroligin-4like, which is localized at inhibitory synapses, human NL4 is mainly expressed at excitatory synapses in the cerebral cortex. The overexpression of NL4 in human embryonic stem cell-derived neurons leads to an increase in the number of excitatory synapses on the one hand, and a significant decrease in synaptic strength on the other, thereby regulating excitatory synaptic transmission in neurons [115].

As for the regulation of synaptic plasticity by NLs, An in vitro study showed that NL1 can be phosphorylated by CaMKII to regulate its function in excitatory synapses [116], as CaMKII plays a key role in the regulation of activity-dependent synaptic plasticity. In addition to this, it was found that knockouts of NL1 in developing neurons of the Xenopus laevis tadpoles retina as well as in isolated hippocampal cultures from newborn NL1 KO mice exhibited a decrease in AMPA receptor current frequency and amplitude as well as AMPA receptor cluster density [24, 117] (Fig. 2). These studies demonstrate the important role of NL1 in the involvement of synaptic plasticity. Using a conditional knockdown approach, researchers determined that NL1 knockdown impaired NMDAR-mediated excitatory postsynaptic currents (NMDAR EPSCs) in the CA1 region of the mouse hippocampus and eliminated NMDAR-dependent LTP [118]. Consistent with this, NL1 KO showed significantly reduced hippocampal synaptosomal expression levels of the AMPA receptor subunit GluA2 and NMDA receptor subunits GluN1, GluN2A, and GluN2B [119]. This decrease in glutamatergic receptors and impaired prominent excitatory transmission both contribute to the diminished synaptic excitatory response. Taken together, NL1 plays an important role in regulating synaptic excitability transmission and synaptic plasticity.

Concerning NL4, unlike mice, human NL4 is predominantly expressed in the cortex and preferentially localized at excitatory postsynapses. Unlike mice, human NL4 is predominantly expressed in the cortex and preferentially localized post-synaptically at excitatory synapses. Overexpression of human NL4 in mouse neurons can specifically alter excitatory synapses [120]. In assessing the effects of NL4 overexpression on human neurons, Marro et al. found that NL4 overexpression increased the number of excitatory synapses while decreasing the frequency of spontaneous excitatory postsynaptic currents (sEPSCs), noting that the amplitude did not decrease as a result (Fig. 2) [115]. This suggests that NL4 overexpression induces the formation of new synapses, but at the same time decreases the proportion of functional synapses [115].

### The function of NLs at inhibitory synapses

As previously described, NL2 is localized to the inhibitory postsynaptic, and previous studies have shown that NL2 overexpression in neuron cultures and mice selectively enhances inhibitory synaptic function, suggesting that NL2 may play a central role in inhibitory synaptic development and function. The total number of inhibitory synapses in NL2KO mice does not appear to be decreased; NL2 affects the postsynaptic protein composition of GABAergic synapses. It was shown that the expression of GABAergic postsynaptic protein complexes (e.g. GABAARs and the scaffolding protein gephyrin) was reduced in NL2KO mice while the expression of presynaptic protein complexes [e.g. vesicular inhibitory amino acid transporter (VIAAT)) appeared to be unaffected [121]. Inhibitory synaptic transmission is often assessed using miniature (mIPSC) or spontaneous (sIPSC)] postsynaptic currents, and in NL2KO mice, not only did inhibitory postsynaptic protein composition change, but mIPSC and sIPSC were also significantly decreased in various brain regions [122–124]. In fact, in most regions, the absence of NL2 would lead to a decrease in the amplitude of mIPSC, and this decrease would imply either a decrease in the function of inhibitory synaptic GABAergic postsynaptic GABA receptors or a decrease in their number. It is puzzling that along with the decrease in mIPSC amplitude there is also a decrease in mIPSC frequency, but mIPSC frequency is generally used to respond to changes in presynaptic GABA release levels at GABAergic synapses, whereas NL2 deletion mainly affects postsynaptic protein composition. It is puzzling that along with the decrease in mIPSC amplitude there is also a decrease in mIPSC frequency, but mIPSC frequency is generally used to respond to changes in presynaptic GABA release levels at GABAergic synapses, whereas NL2 deletion mainly affects postsynaptic protein composition. In addition, the binding of neuroligins and his receptor neurexins mediated trans-synaptic signaling may also have some effect on presynaptic GABA release, which in turn reduces the frequency of mIPSC. These are two possible mechanisms for the reduction of mIPSC frequency, and their contributions to mIPSC frequency have not been elucidated, but they may not be opposed to each other, which of course needs to be further explored.

In addition to NL2, NL3 can also be localized after inhibitory synapses, and many studies exist on the role of NL3 on GABA receptor-mediated inhibitory synapses like the decrease in amplitude and frequency of mIPSC caused by NL2 deletion, in CA1 pyramidal neurons, total or conditional knockout of the NL3 gene only leads to an increase in the frequency of mIPSC without affecting its amplitude [118, 125]. Compared with NL3 KO mice, NL3 R451C-knockin mice carrying R451C replacement for human autism mutations also showed a strong synaptic phenotype but failed to find significant changes in NL3 knockout mice. Földy et al. used paired recordings in mice carrying these mutations to measure synaptic transmission at GABAergic synapses formed on pyramidal neurons by basket cells expressing hippocampal microstrip and cholecystokinin. They found that disruption of obligatory endogenous cannabinoid signaling at cannabinoid CB1 receptors expressed in Cck+ inhibitory synapses selectively enhanced both Cck+ synapses in NL3KO mice and synaptic transmission at Cck+ inhibitory synapses in NL3-R451C KI mice [126]. However, the importance of NL3 for this endogenous cannabinoid transmission seems to be limited to early development, as the NL3 conditional KO mice at P21 did not cause changes in mIPSC amplitude and frequency [118].

Inhibitory synaptic transmission in other regions of the brain is also influenced by NL3 function. Nlgn3-R451C KI mice have altered synaptic activity in the basolateral amygdala and exhibit reduced mIPSC amplitude [127]. Not only that, impaired endogenous cannabinoid signaling at CB1-expressing Cck+ synapses in Nlgn3-R451C KI mice resulted in elevated VITTA expression. At the same time, inhibitory synaptic transmission is significantly increased in Nlgn3-R451C KI mice [128].

## Neuroligins in the ‘synaptopathy’

In recent years, the concept of ‘synaptopathy’ has been extended from neurodegenerative and neurological disorders to psychiatric diseases.

Disruption of synaptic structure and function is a major determinant of psychiatric diseases. NL is associated with a variety of neurological disorders including Alzheimer’s disease, schizophrenia, ASD, etc. It is not surprising to see that loss of glial neuroligin function disrupts synaptic structure and function, suggesting the involvement of glial neuroligin in the pathogenesis of these diseases. Therefore, a full understanding of how glial neuroligin affects the structure and function of synapses facilitates a deeper understanding of the complex mechanisms of neurological diseases.

### Neuroligins and Alzheimer’s disease

Alzheimer’s disease (AD) is the leading cause of dementia in the elderly population, and AD is characterized by severe cognitive deficits, including memory loss and language impairment. In addition to cognitive deficits, neuropsychiatric symptoms such as depression, apathy, and hallucinations are often seen in patients with AD [129, 130]. As a neurodegenerative disorder, the key pathological features of AD include neuronal loss and cellular dysfunction. Neuronal loss and synaptic loss are the main causes of cognitive impairment in AD patients, which also fully demonstrates the importance of synaptic alterations in the pathogenesis of AD [131]. Moreover, in the early stages of the disease, there is significantly more synaptic loss than neuronal loss [131–133], which is a strong indication that synaptic dysfunction can be a major factor in the cognitive decline of AD [134]. In the AD mouse model, synaptic dysfunction occurs in the first phase [135, 136] and disruption of synaptic connections will in turn lead to neuronal dysfunction resulting in cognitive deficits [134].

Neuroligins mediate basic synaptic functions. However, NL gene mutations have been found in patients with autism and other neurological disorders [137, 138], suggesting that neuroligins may be closely associated with cognitive impairment. The researchers found that neuroligin-1 knockout mice exhibited increased repetitive grooming movements similar to those observed in individuals with autism, suggesting that such repetitive movements may be associated with autism [67]. Not only that, but Neuroligin-1 knockout would also result in a reduced NMDA/AMPA ratio at cortico-striatal synapses in mice [67]. Abnormalities in this ratio can also be seen in AD patients and are closely associated with the critical regulatory role of NL1 on synapses. Thus this evidence suggests that NL1 is most likely involved in the pathogenesis of AD. When neuroligin-1 protein is overexpressed, the regulation of the ratio of excitatory to inhibitory synapses by NL and NRX is altered, resulting in defective memory acquisition, increased maturation of excitatory synapses, and impaired memory formation, synaptic plasticity, and impaired learning in mice [10]. In mice, inhibition of neuroligin-1 would reduce NMDA receptor-mediated currents and prevent the expression of LTP. Specifically, neuroligin-1 inhibition would reduce LTP expression by decreasing NMDA receptor-mediated currents, thereby blocking the storage of associative fear memory [9]. NMDA receptor-mediated synaptic transmission underlies the formation of long-term memory in animals, and the above results suggest that sustained expression of NL1 is necessary to maintain NMDAR-mediated synaptic transmission. Similarly, knockdown of NL1 in mice would result in impaired hippocampal long-term potentiation, causing mice to exhibit deficits in spatial learning and memory [67].

By overexpressing NL2, the researchers found that small changes in NL2 expression could lead to a larger synaptic contact area and an enlarged pool of synaptic vesicles in the frontal cortex, as well as an overall decrease in the excitation and inhibition (E/I) ratio [139]. These animals also exhibit stereotyped jumping behavior, anxiety, impaired social interactions, and an increased incidence of spike-wave discharges [139]. Excitation levels in the brain are controlled by inhibitory signals exerted primarily by GABA neurons, and this E/I imbalance and the behavioral changes associated with neurodevelopmental disorders may form the basis of neurodevelopmental disorders.

Neuroligin-3 mutant mice with an arginineto-cysteine substitution at amino acid #451 (NL3 R451C) exhibit moderately impaired social behavior, enhanced water maze learning ability, and increased synaptic inhibition in the somatosensory cortex. This is thought to be caused by increased inhibitory synaptic transmission [128].

Acetylcholinesterase is the first synaptic protein described to interact with Aβ, and the sequence of the extracellular structural domain of the NLs family is homologous to cholinesterase, making NLs a candidate synaptic protein that may affect intracellular Aβ deposition [140]. Dinamarca et al. used intrinsic fluorescence and surface plasmon resonance to find that Aβ binds to the extracellular domain of NL-1 with a K(d) in the nanomolar range. In addition, the interaction of NL-1 with Aβ increased the formation of Aβ oligomers, suggesting that this interaction may trigger the targeting of Aβ oligomers to the postsynaptic region of excitatory synapses, which may lead to synaptotoxicity and degeneration of AD [141]. ApoE4 is the major known genetic risk factor for AD, accounting for more than 95% of AD cases [142]. Zhong et al. found a reduced density of NL1-immunoreactive postsynaptic terminals in a mouse model (Arg-61 apoE mice) expressing a variant of the APOE protein showing all apoE4-specific structural properties, implying the presence of this postsynaptic defect in this mouse model [143]. Furthermore, in the brain and neuronal cultures, ADAM10, the major shedding enzyme of NL1, catalyzed the cleavage of the extracellular structural domain of NL1, followed by cleavage of the car boxy-terminus region of NL1 by γ-secretase to release its intracellular structural domain fragments. Not only that, this NL1 cleavage is also regulated by neuronal excitatory activity, as an increase in NL1 shedding is observed with the addition of both NMDA and β-neurexin to the culture medium. This NL1 shedding leads to a decrease in the number of dendritic spines in neuronal cultures [144]. The extracellular environment of the AD brain may be excitotoxic due to enhancement of glutamate receptors by neuroligin-1. This glutamate shedding via NMDAR-mediated regulation of NL1 may have implications for neuronal activity. Similarly, postsynaptic cleavage of NL1 could affect the stability of presynaptic NRX1β and thus the overall synaptic function [145].

In rodents, amyloidogenic fiber-induced neuroinflammation enhances the activity of the HDAC2-MeCP2 corepressor complex and decreases NL1 expression, leading to hippocampal glutamatergic dysfunction and cerebral ischemia, which may be a potential cause of amyloid-induced memory deficits [146]. The potential role of NLs for AD is expected to translate into effective pharmacological interventions for AD patients to reduce neuroinflammation and neurodegenerative degeneration.

### Neuroligins and Autism Spectrum Disorder

Autism is a broad cognitive disorder characterized by impairments in social interactions, such as daily communication, social interaction, and play, and can be accompanied by stereotypical patterns of behavior [147, 148]. Autism has an extremely strong genetic background, which manifests itself in childhood. The symptoms of ASD are part of a variety of neurological disorders such as fragile X syndrome and Rett syndrome [149–151]. Usually, ASD is closely associated with mental retardation, but individuals with ASD occasionally show enhanced cognitive abilities, which is known as the ‘autistic savant syndrome’ [152]. The inheritance of ASD is highly complex and heterogeneous, involving not only the number of genes involved but also the nature of the genetic variation [153]. A few cases of idiopathic ASD are associated with mutations in individual genes, including those encoding neuroligins and their associated proteins [154].

Researchers found missense and non-missense mutations in NL3 and NL4 in some human ASD patients [12, 155, 156]. Genetic analysis of rare ASD variants has also shown a potential association with NL3 and NL4 mutations in humans [12, 157].

Several point mutations in the NL are associated with ASD, most of which result in substitution localization on extracellular protein structural domains, and only a few localize on intracellular structural domains.

To this point, five rare NL1 mutations associated with ASD have been identified, corresponding to substitutions in P89L, T90I, L269P, G297E, and H795Y [158]. Among these, only H795Y is localized within the cytoplasmic structural domain, while the remaining four substitutions are located in the extracellular cholinesterase structural domain and play a role in altering the protein structure. The stability of the protein surface-rich proline loop was mainly affected by the P89L and T90I substitutions [40, 159]. Computerized prediction of the pathogenic role of these five NL1 variants in patients with ASD using CADD [160], SIFT [161], PolyPhen2 [162], MutationTaster [163], LRT [164], Mutation Assessmentor [165], and FATHMM [166], according to the predicted results, these mutants can be classified into two pathogenic variants, high-risk variants (P89L, L269P, and G297E) and low-risk variants (H795Y and T90I). Unlike NL1 wild-type mice, high-risk mutants cause altered protein transport. May lead to misfolded proteins, leaving these mutated molecules stranded in the endoplasmic reticulum (ER) [158]. The two low-risk variants were not associated with changes in subcellular localization, but H795YS had a higher susceptibility to hydrolytic cleavage and degradation of the protein thereby reducing its expression level [158]. P89L NL1 variant knock-in (KI) mice can exhibit abnormal social behavior and impaired spatial memory [158], whereas NL1 KO mice exhibit spatial memory deficits and increased repetitive behavior [9, 67, 119].

An in vivo study showed that NL2 deficiency leads to impaired inhibitory synaptic function without affecting its number and that this reduced synaptic function is associated with a range of behavioral phenotypes including a significant increase in anxiety-like behavior, and reduced pain sensitivity, and motor coordination [167]. Sun et al. identified six rare missense point mutations including R215H, V510M, R621H, A637T, P800L, and A819S by systematically screening for mutations in NL2 exons and promoter regions in a cohort of schizophrenic patients [137], where R215H,6V510M are located in the NL2 extracellular cholinesterase-like protein structural domain, while R621H and A637T are in the extracellular stalk structural domain and intracellular WW binding domain. The R215H variant is retained in the endoplasmic reticulum and has reduced expression at the plasma membrane, resulting in a defective GABAergic synapse due to the inability to transport it to the cell membrane. Not only that, the aggregation of the variant expressing R215H with NRX1-β-expressing cells was significantly reduced and the variant failed to induce the formation of GABAergic synapses in co-culture experiments. This may be due to the retention of NL2 in the endoplasmic reticulum, preventing the cell surface and extracellular export of NL2 from binding to NRX1-β [137]. NL2 R215H KI homozygous mice exhibit several behavioral abnormalities similar to those of psychiatric patients carrying the NL2 mutation such as growth retardation, anxiety-like behavior, impaired spatial learning, and memory [168].

The Arg residue at position 451 in NL3 is highly conserved in cholinesterase-like proteins[169], and the R451C substitution impairs NL3 folding and/or dimerization and transport to the cell surface, leading to retention of the endoplasmic reticulum and degradation via the proteasome [170–172]. In addition to this, this substitution would lead to defective transport of NL3, leaving the protein stranded on the endoplasmic reticulum, which in turn leads to reduced delivery of NL3 to the cell surface. Even if a small fraction of the protein reaches the cell membrane surface, its binding activity to NRX1-β has been significantly reduced [173]. NL3-KO mice exhibited behaviors similar to human ASD symptoms such as reduced vocalizations and social memory deficits [174], and functional alterations in different brain regions not found in NL3 KO mice [125, 128]. R451C NL3 KI mice also exhibit autism-like genotypes in cognitive and social Tasks [128, 175]. Although functional alterations in different brain regions have not been found in NL3 KO mice, several brain regions in R451C NL3 KI mice have smaller volumes of gray matter structures such as the hippocampus, striatum, and thalamus than wild mice, while the volume of white matter structures such as the cerebral peduncle, corpus callosum, fornix/fimbria, and internal capsule was significantly reduced, and this reduction appeared to be due to a decrease in the number of axons or less mature axons [176]. R451C NL3 KI MICE also significantly increase AMPAR-mediated excitatory synaptic transmission in the hippocampal CA1 region and markedly alter the kinetics of NMDA receptor-mediated synaptic responses [125].

In conclusion, studies of R451C NL3 KI mice showed social behavior deficits [177], increased repetitive, stereotypic behavior [178], and disruption of excitatory and inhibitory synaptic balance [128]. All these phenomena are closely related to the abnormal circuit-dependent synaptic efficiency caused by ASD-associated NL3 mutations, and the circuit-specific function of NL3 and its potential molecular mechanisms important in ASD have been reviewed in detail by Uchigashima et al. [38]. These suggest a mechanism for NL3 function in controlling the behavioral phenotype of ASD.

Similar to NL3, NL4 KO mice can exhibit highly selective deficits in social communication and interaction similar to those exhibited by human ASD. Not only that but the brain volume of NL4 KO mice was also reduced relative to WT mice [174, 179]. Genetic findings also support the conclusions of this animal study, and these results illustrate the relevance of NL4 gene mutations to ASD as well as cognitive impairment [156].

There is no direct evidence for the involvement of glial cell NL in the pathogenesis of ASD. Valproic acid (VPA) is an anticonvulsant drug widely used in epilepsy, and its anticonvulsant effect may be produced by modulating the synaptic excitation/inhibition balance [180]. Some studies have shown that VPA use by pregnant women will increase the incidence of ASD in them [181]. Wang et al. demonstrated in a primary rat neuron, astrocyte, and glial cell co-culture system that VPA treatment significantly upregulated NL1 transcription and that this upregulation was only seen in astrocytes but not in neuronal cells [182]. From this, we can speculate that NRX–NL-mediated cross-cellular contacts between astrocytes and neurons may be compromised. Whether astrocytes are treated with VPA to affect functions other than synaptic excitation/inhibition balance is still unknown.

### Neuroligins and schizophrenia

Schizophrenia is a heterogeneous syndrome rather than a single defining sign or symptom [183]. Symptoms of schizophrenia include positive symptoms (delusions, hallucinations) and negative symptoms (decreased emotional expression and response, decreased interpersonal involvement, decreased speech production, and apathy). These symptoms usually appear in late adolescence or early adulthood. Disruption of synaptic connectivity is thought to be a major pathological driver of schizophrenia [184]. Schizophrenia is highly heritable, with the results of a meta-analysis showing a probability of inheritance of roughly 70–80% [185]. The genetics of schizophrenia are complex and analysis of genetic data is difficult. In patients with schizophrenia, researchers have identified mutations in the NL2 [137] and NL4 [186] genes.

An in vivo study showed that NL2 deficiency leads to impaired inhibitory synaptic function without affecting its number and that this reduced synaptic function is associated with a range of behavioral phenotypes including a significant increase in anxiety-like behavior, and reduced pain sensitivity, and motor coordination [167]. Sun et al. identified six rare missense point mutations including R215H, V510M, R621H, A637T, P800L, and A819S by systematically screening for mutations in NL2 exons and promoter regions in a cohort of schizophrenic patients [137], where R215H and 6V510M are located in the NL2 extracellular cholinesterase-like protein structural domain, while R621H and A637T are in the extracellular stalk structural domain and intracellular WW binding domain. The R215H variant is retained in the endoplasmic reticulum and has reduced expression at the plasma membrane, resulting in a defective GABAergic synapse due to the inability to transport it to the cell membrane. Not only that, the aggregation of the variant expressing R215H with cells expressed NRX1β was significantly reduced and the variant failed to induce the formation of GABAergic synapses in co-culture experiments. This may be due to the retention of NL2 in the endoplasmic reticulum, preventing the cell surface and extracellular export of NL2 from binding to NRX1β [137]. NL2 R215H KI homozygous mice exhibit several behavioral abnormalities similar to those of schizophrenia carrying the NL2 mutation such as growth retardation, anxiety-like behavior, impaired spatial learning, and memory [168]. In addition, disruption of GABAergic synaptic transmission in schizophrenic patients would lead to deficits in inhibitory circuit function, which would also result in memory impairment [187]. Studies of NL2 in animal models of schizophrenia following postnatal administration of NMDA receptor antagonists revealed that only NL2 protein levels in the medial prefrontal cortex of the adolescent rat brain were altered, while adult rats did not appear to be affected by NL2 protein expression levels [188]. NL2-R215H knock-in mice exhibit reduced inhibitory synaptic proteins, corresponding defects in inhibitory synaptic transmission, and schizophrenic-like behaviors including anxiety and stress responses [168, 189]. All of these results nicely illustrate that changes in NL2 protein levels may lead to inhibitory synaptic dysgenesis, such as the defective GABAergic transmission observed in the brains of schizophrenic patients, and that this dysgenesis is most likely to occur during adolescence.

In terms of glial neuroligins, although there are no direct studies for the time being to suggest that glial NLs are involved in the pathogenesis of schizophrenia, a study using mice chimeric with human patient-derived glial progenitor cells found a significant downregulation of NL3 in chimeric glial progenitors compared to controls [190]. In addition, chimeric mice exhibit impaired astrocyte differentiation and reduced morphological complexity [190]. Having previously detailed that astrocyte NL regulates astrocyte morphogenesis and the interaction between astrocytes and synapses, it is not difficult to link the abnormal behavior of schizophrenia to astrocyte NLs.

## Discussion

In this review, we attempt to address the important roles of neuronal neuroligins and glial neuroligins in synaptic development, synaptic transmission, synaptic plasticity, and neuropsychiatric disorders from both perspectives. Although the process of synapse formation has not been clearly described in decades of research, there is no doubt that CAMs, represented by neuroligins, play a crucial role in the process of synapse formation. Given that synaptic connections ensure the proper functioning of neural circuits in the brain and that structural and functional dysfunctions of synapses are important determinants of psychiatric disorders, it is important to analyze the basic mechanisms of synaptic development. Understanding these mechanisms not only helps us to understand how the brain works but also provides insight into psychiatric or neurodegenerative disorders. However, for the current study, we still have many questions to explore the role of neuroligins in the development of the central nervous system in more depth.

First, as for the basic mechanism of glial NL, most studies still focus on its role in binding with its typical partner NRX. Recent studies have found that NL does not work only through binding to NRX. NL can not only participate in the regulation of synapses through its specific extracellular domain [68], but also directly bind to glutamate receptors to change synaptic strength. Not only that, the binding of NL3 to its atypical partners such as PTPδ can undergo trans-synaptic interactions mediating noncanonical NL3-PTPδ signaling [55]. Since the composition of the cytoplasmic membrane is not uniform across cell types, for example, the scaffolding molecule PSD-95 is present only as a major component of the excitatory postsynaptic membrane. Therefore, there must be differences in the intercellular Signal Transduction between neuronal neuroligin and glial neuroligin. The above-mentioned role played by NL independently of NRX gives us new insights. Whether glial cells (whose plasma membrane is different from that of neurons) can also participate in the regulation of synaptic function through these pathways. There is limited direct research on the signaling mechanisms of glial NL between cells, and the role of glial cells in the CNS determines the importance of this signaling mechanism of glial NL between cells for the maintenance of CNS function. In the future, studies on the communication between glial NL and neurons are still of great interest.

Second, our understanding of the structure of neuroligins is actually more than its physiological role, which fully illustrates that it is much more difficult to understand the physiological function of proteins than to understand their molecular structure. We need to further understand the specific role of glial neuroligins or neuronal neuroligins at the synapse. For example, the specific mechanism of E/I ratio imbalance due to NLs and how NLs mediate the transduction of synaptic transmission signals remain a major challenge for now. This will have a direct impact on our understanding of neural circuits.

Third, although recently Stogsdill et al. linked astrocyte morphogenesis to synaptogenesis via astrocyte NLs and showed the possibility that synaptic pathological changes associated with NLs mutations depend on or even originate from astrocyte dysfunction [98]. However, there are still the following issues that need to be studied. 1. What downstream pathways exist for astrocyte NLs to regulate astrocyte morphogenesis; 2. Whether bidirectional signaling via astrocyte NLs-mediated astrocyte-neuron adhesion directly regulates synapse formation and function, and what their specific mechanisms are; 3. The specific mechanisms by which astrocyte NLs control synaptic connections.

Fourth, in addition to astrocytes, we summarize the role of oligodendrocyte NLs in the central nervous system. Proctor et al. demonstrated that oligodendrocyte NLs play an important role in oligodendrocyte differentiation as well as myelin formation [64], but how oligodendrocyte NLs promote oligodendrocyte differentiation as well as myelin formation is unclear.

Finally, a large number of existing studies have focused on the indispensable role of neuronal NLs in neurodevelopmental diseases such as Schizophrenia, ASD, and AD, but little is known about the role of glial NLs in related diseases. Glial NLs are also abundantly expressed in the CNS and influence synaptic development at all times, affecting the function of neural circuits in the brain. And circuit dysfunction underlies many neurodevelopmental disorders, which means that exploring the role of glial cell NLs in neurodevelopmental disorders is relevant and beneficial to one’s understanding of these disorders.

In conclusion, future individualized studies of different cell types expressing NLs genes are still needed to assess the similarities and differences in the role of NLs expressed by each cell type. The determination of synaptic transmission, synaptic structure, and the location of NLs and synapses can be performed with the help of CRISPR tools. Both fundamental mechanistic analysis of NLs in synaptic development and exhaustive molecular structural analysis of NLs are beneficial to the understanding of normal brain functioning as well as brain diseases.

## Data Availability

Not applicable.
